# Rotational alignment of femoral component with different methods in total knee arthroplasty: a randomized, controlled trial

**DOI:** 10.1186/s12891-017-1574-5

**Published:** 2017-05-25

**Authors:** Joon Kyu Lee, Sahnghoon Lee, Sae Hyung Chun, Ki Tae Kim, Myung Chul Lee

**Affiliations:** 1 Department of Orthopaedic Surgery, Hallym University Sacred Heart Hospital, 22 Gwanpyeong-ro, 170beon-gil, Dongan-gu, Anyang-si, Gyeonggi-do 431-796 Korea; 2 Department of Orthopaedic Surgery, Seoul National University Hospital, 101 Daehang-ro, Jongno-gu, Seoul, 110-744 Korea

**Keywords:** Femoral component rotation, Gap technique, Measured resection technique, Tensor device

## Abstract

**Background:**

Femoral component rotation (FCR) is one of the most important factors in total knee arthroplasty. In this prospective study, we used three different techniques for FCR and analyzed their accuracy with postoperative axial computed tomography (CT) images. We also evaluated effect of FCR to clinical outcome.

**Methods:**

One hundred sixty-five patients were randomly allocated into three groups. In the measured resection group, FCR was set by externally rotating the axis 3° off the posterior femoral condylar axis. In the tensor group, a gap-tensioning device set at 20 lbf was used. In the block group, spacer blocks of various thicknesses were used. The FCR angle (FCRa) was measured on postoperative axial CT as an angle between the clinical transepicondylar and posterior condylar axes of the femoral component. Outliers were defined as FCRas deviated more than 3° either internally or externally. Postoperative 2 year clinical scores and knee range of motion were checked.

**Results:**

The tensor group had significantly better positioning of the femoral component to the neutral position compared with the measured resection group and the block group (mean FCRa: internal rotation 1.79, 0.43 and 2.63°, respectively, *p* < 0.001). The outliers were also least frequent in the tensor group (35, 16 and 40%, respectively, *p* = 0.02). There were no significant differences in postoperative 2 year clinical results among groups.

**Conclusions:**

Gap technique with a 20-lbf tensor device was the most accurate and precise method for obtaining adequate FCR. Measured resection with 3° external rotation and gap technique with blocks could lead to internal rotation of the femoral component. Postoperative 2 year clinical results were not significantly different among groups with different techniques for FCR.

**Trial registration:**

The study was registered in the Clinical Research information Service (trial number: KCT0000129) in Korea. Registration date is 23^rd^ of June, 2011.

## Background

Establishing adequate femoral component rotation (FCR) is important in total knee arthroplasty (TKA), and it is widely accepted through many studies. Most surgeons agree that the femoral component should be rotated externally as emphasized by Mochizuki more than 30 years ago [1]. Various patellofemoral complications are observed when the femoral components are rotated internally, such as lateral tilting, subluxation and dislocation of the patella and patellar maltracking [2]. Increased lateral flexion laxity is associated with increased internal rotation of the femoral component and a less favorable clinical outcome [3]. On the contrary, excessive external rotation of the femoral component will increase the medial flexion gap and could lead to symptomatic flexion instability. Combined internal malrotation of the femoral and tibial component is also a significant factor in the development of anterior knee pain after TKA [4]. Patellofemoral problem, instability, polyethylene wear, osteolysis, aseptic loosening and infection are major causes of early failures in TKA [5].

Two techniques are generally accepted for soft tissue balancing and determining FCR, which are measured resection and gap techniques [6, 7]. An external rotation of 3° off the posterior femoral condylar axis is considered to be satisfactory and generally accepted in the measured resection technique [8, 9]. However, several other methods have been proposed in an effort to increase its accuracy. Many studies have reported that the transepicondylar axis is more reliable in a typical varus knee with medial tibiofemoral arthritis [10–13]. Two methods are used to determine the flexion gap and femoral component position in axial plane in the gap technique. The tensor device is commonly used to achieve symmetric gap. It is still unclear how much force is appropriate for distraction using the tensor device, and surgeons usually determine the device tension through their experience [14, 15]. Alternatively, gap blocks of various thickness can be used to perform the gap technique.

The primary purpose of this study was to find whether different methods may result in different outcomes in FCR accuracy and outlier frequency. The secondary purpose was to identify the effect of FCR to the clinical outcome. The hypotheses were that there would be significant differences in FCR accuracy among techniques and FCR would affect clinical outcome significantly. We prospectively performed TKAs with three different methods and analyzed its FCR accuracy by measuring the degree of FCR to the clinical transepicondylar axis (cTEA) on the postoperative axial computed tomography (CT) images. Postoperative 2 year clinical scores and knee range of motion were evaluated to check the effect of FCR to the clinical outcome.

## Methods

**Fig. 1:**
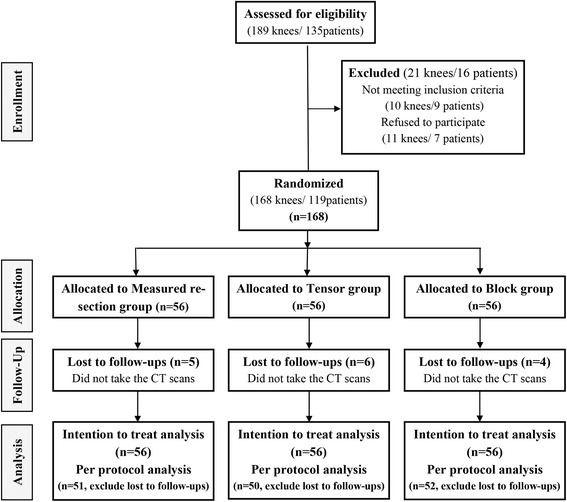
A CONSORT (Consolidated Standards of Reporting Trials) flow diagram of the study

Consecutive patients, who were scheduled to undergo primary TKAs, were enrolled prospectively between June 2011 and August 2012. In 132 patients, TKAs were performed on 189 knees during the enrollment period of this study. Patients with a diagnosis other than primary osteoarthritis or valgus deformity of the knee and those who refused to participate were excluded. After written informed consents were obtained, 168 knees in 119 patients were assigned to one of the three groups. Block randomization using sealed envelopes was carried out in the operation room. CT scans were performed approximately 3 months after surgery, and 15 knees in 12 patients were lost to follow-up because these patients did not undergo CT scanning. Consequently, 153 knees in 107 patients were analyzed in this study (Fig. 1). No inter-group differences were evident in preoperative demographics and clinical status (Table 1).

**Table 1:** Comparison of pre-operative demographics and clinical status among the groups

	Measured resection (*n* = 51)	Tensor (*n* = 50)	Block (*n* = 52)	*p*-value
Gender (M/F)	2/49	1/49	2/50	0.828^a^
Age^c^ (year)	68.6 (55–81)	70.8 (56–86)	69.3 (56–78)	0.178^b^
Body mass index^c^ (kg/m^2^)	26.6 (20.8–33.7)	25.6 (20.7–31.2)	25.8 (20.2–33.3)	0.169^b^
Involved knee (Rt./Lt.)	27/24	21/29	25/27	0.545^a^
Range of knee motion^c^ (degree)				
Flexion contracture	9.4 (0–25)	8.8 (0–30)	8.8 (0–20)	0.859^b^
Further flexion	124.7 (100–150)	125.9 (90–150)	125.5 (40–150)	0.937^b^
Total range of motion	115.3 (80–145)	117.1 (60–145)	116.7 (35–145)	0.889^b^
Tibiofemoral angle (degree)	Varus 4.5 (varus 24 - valgus 7)	Varus 2.8 (varus 19 - valgus 6)	Varus 2.9 (varus 18 - valgus 7)	0.225^b^
KS score^c^ (points)				
Knee	48.6 (19–90)	45.1 (6–74)	46.6 (24–71)	0.259^b^
Function	42.7 (0–86)	39.7 (0–71)	39.4 (2–71)	0.163^b^
HSS score (points)	62.2 (38–84)	60.9 (27–82)	61.9 (43–78)	0.802^b^
WOMAC score (points)	52.8 (19–96)	58.3 (17–96)	55.8 (17–91)	0.094^b^

Abbreviations: *KS* Knee Society, *HSS* Hospital for Special Surgery, *WOMAC* Western Ontario and McMaster Universities Osteoarthritis Index (LK 3.1 version)

^a^Chi-square test

^b^Mixed model for adjustment of auto-correlation

^c^The values are given as the mean and the range in parenthesis

**Fig. 2:**
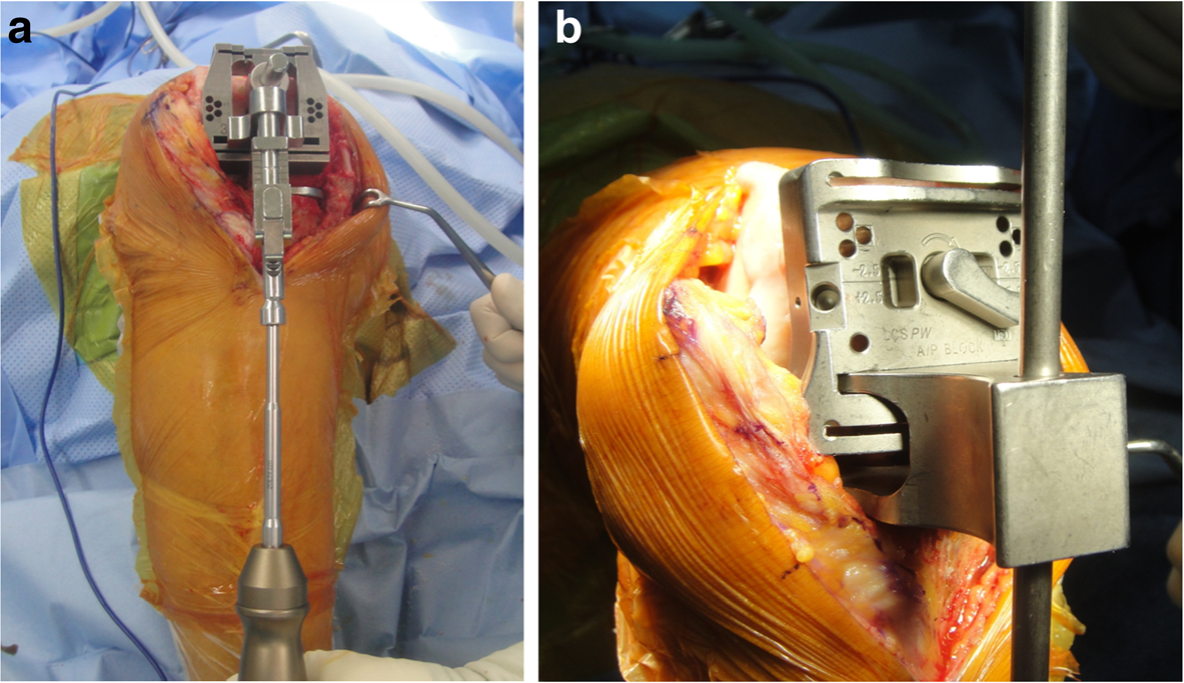
Devices used during the surgery to set femoral component rotation: **a** the tensor device set by 20 lbf which was used in the tensor group, **b** the gap block which was used in the block group

All surgical procedures were performed by a single experienced surgeon (*). We used the P.F.C Sigma RP-F (DePuy Orthopaedics, Leeds, United Kingdom), Buechel-Pappas TKA system (Endotec, Orlando, Florida, USA) and Low Contact Stress TKA system (DePuy, Warsaw, IN, USA) for the measured resection, tensor and block groups, respectively. A medial parapatellar arthrotomy was used, and both cruciate ligaments were resected. In the measured resection group, we resected the distal femur and proximal tibia and subsequently determined the FCR using the sizing guide instrument. The FCR axis was set by externally rotating the axis 3° off the posterior femoral condylar axis. In the tensor group, we cut the proximal tibia, then used the tensor device to determine component rotation in which the joint distraction force was set at 20 lbf (89 N) (Fig. 2a). After that, we resected the anterior and posterior femur, and then, the distal femur. In the block group, the procedure was the same as that in the tensor group, but instead of using the tensor device, gap blocks with 10, 12.5, 15 and 17.5-mm thicknesses were used (Fig. 2b). Most patellae were resurfaced. However, normal-shaped patellae with thickness less than 20 mm or relatively good cartilage status (International Cartilage Repair Society grade 0 or 1) were retained selectively. All prostheses were fixed with cement.

**Fig. 3:**
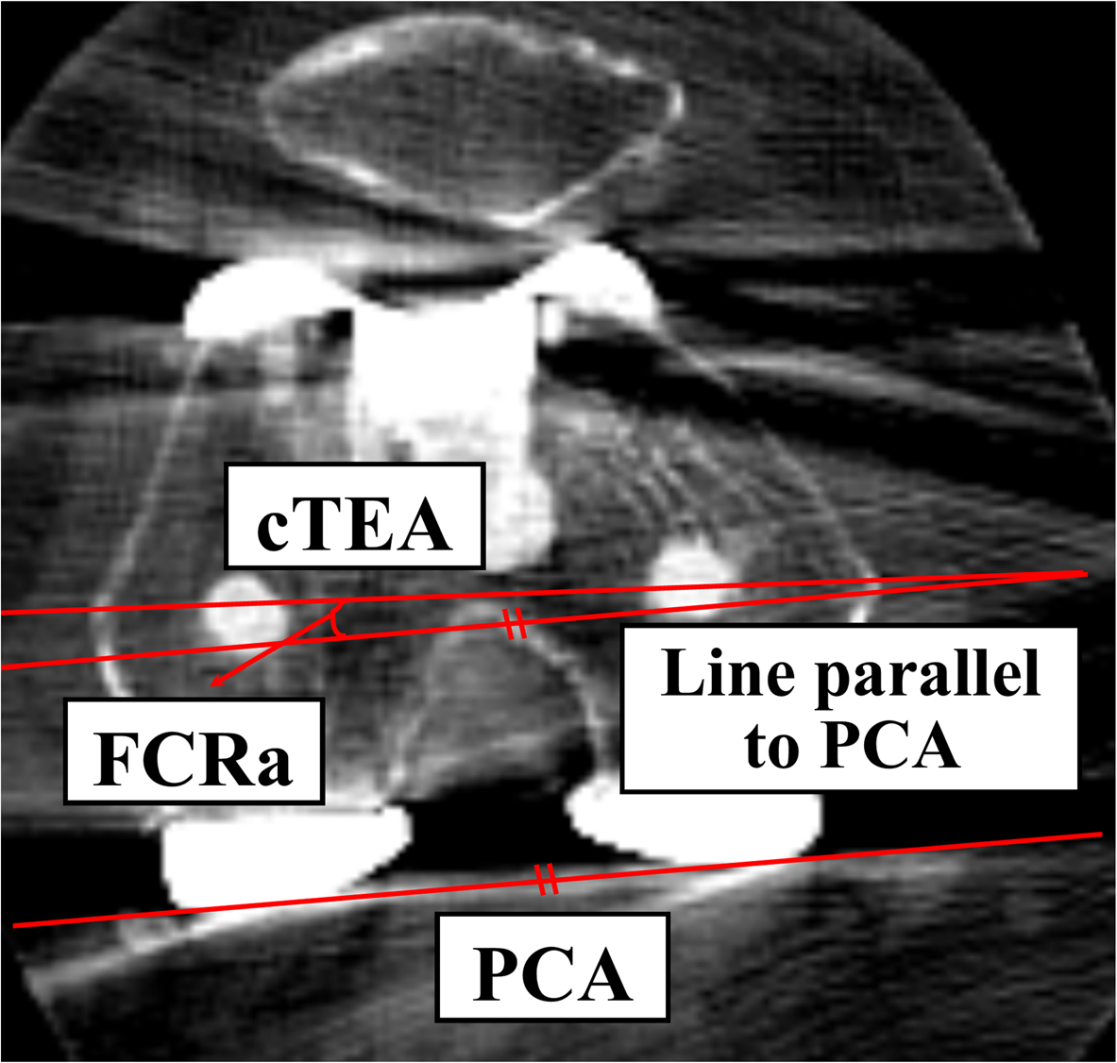
Angle on the axial CT image manipulated with use of the OnDemand3D program (Cybermed) to measure femoral component rotation (Tensor group). cTEA, clinical transepicondylar axis; PCA, posterior condylar axis; FCRa, Femoral component rotation angle, the angle between cTEA and PCA of the femoral component

Using CT scans (Siemens Somatom; Siemens Medical Solutions, Malvern, PA, USA), 1-mm thickness axial images were obtained. We measured the angle between the cTEA and the posterior condylar axis (PCA) of the femoral component (FCR angle, FCRa) using the OnDemand3D program (CyberMed, Seoul, Korea) (Fig. 3).

We calculated the mean value of the FCRa and the frequency of outlier, which was defined as the deviation of more than 3° either internally or externally, in each group [6, 16]. Clinical effect of the FCR was evaluated with clinical scores (Knee society scores, Hospital for special surgery score and WOMAC score) and knee range of motion at 2 years postoperatively.

### Statistical analysis

A priori sample size analysis using G*Power program version 3.1.2 showed that 50 cases per group were required to detect a statistical difference in the component rotation among three groups with a 1° precision, (α = 0.05, β = 0.8). The results were evaluated only in the per-protocol analyses after excluding patients lost to follow-ups rather than applying an intention-to-treat protocol. The Kolmogorov-Smirnov test was used to check the data for normality in continuous variables. The inter-group differences were determined using mixed model for adjustment of auto-correlation and Pearson’s chi-square test with posthoc tests for continuous and categorical variables, respectively. All statistical analyses were performed using two-tailed test, and significance was accepted for *p* value of <0.05. Two of authors (* and *) measured the FCRa on axial CT images twice, with an interval of 2 weeks between measurements. The intra- and interobserver reliabilities in measurements were verified by measuring agreement with kappa statistics [17].

## Results

The mean FCRa was −1.79° ± 2.25°, −0.43° ± 2.36°, and −2.63° ± 2.50° in the measured resection, tensor and block groups (‘-’ means internal rotation of the femoral component), respectively. The tensor group had significantly better positioning of the femoral component to the neutral position compared with the measured resection and block groups (*p* < 0.001). The outliers were also least frequent in the tensor group (35, 16 and 40% in the measured resection, tensor and block groups, respectively, *p* = 0.02). The measured resection group and the block group showed internally rotated positioning of the femoral component, and no significant difference was found between the two groups. The outlier cases were also similar between the two groups (Table 2).

**Table 2:** Comparison of the femoral component rotation

		Measured resection (*n* = 51)	Tensor (*n* = 50)	Block (*n* = 52)	*p*-value
Femoral component rotation^c,d^ (degree)	Mean	−1.79	−0.43	−2.63	0.0001^a^
	Range	−6.9 ~ 3.4	−5.5 ~ 6.1	−10.8 ~ 1.5	
	95% confidence interval	−2.43 ~ −1.16	−1.10 ~ 0.24	−3.32 ~ −1.93	
Outlier^e^	Total	18 (35%)	8 (16%)	21 (40%)	0.020^b^
	>3° IR	16	5	21	
	>3° ER	2	3	0	

Comparison between Measured resection group and Tensor group; *p*-value 0.0063^a^ Comparison between Tensor group and Block group; *p*-value <0.0001^a^ Comparison between Measured resection group and Block group; *p*-value 0.0691^a^

Femoral component rotation: angle between clinical transepicondylar axis and posterior condylar axis of femoral component

Outlier: Femoral component rotation with more than 3° of external rotation or internal rotation

Abbreviations: *IR* internal rotation, *ER* external rotation

^a^Mixed model for adjustment of auto-correlation

^b^Chi-square test

^c^Subgroup analysis of femoral component rotation angle among groups

^d^+ : external rotation, − : internal rotation

^e^The values are given as the number of cases with percentage in parenthesis

There were no significant differences in postoperative 2 year clinical results among groups, although the tensor group had slightly better results compared to two other groups (Table 3).

**Table 3:** Comparison of postoperative clinical scores and knee range of motion among the groups

	Measured resection (*n* = 45)	Tensor (*n* = 48)	Block (*n* = 49)	*p*-value
Range of knee motion^b^ (degree)	127.7 (90–145)	128.0 (100–145)	125.9 (95–145)	0.400^a^
KS score^b^ (points)				
Knee	95.2 (84–100)	96.2 (84–100)	95.9 (89–100)	0.783^a^
Function	85.3 (61–100)	85.0 (64–100)	83.4 (61–100)	0.760^a^
HSS score (points)	91.7 (82–99)	92.0 (84–99)	91.8 (84–98)	0.899^a^
WOMAC score (points)	12.1 (1–24)	11.4 (1–27)	11.9 (2–27)	0.831^a^

Abbreviations: *KS* Knee Society, *HSS* Hospital for Special Surgery, *WOMAC* Western Ontario and McMaster Universities Osteoarthritis Index (LK 3.1 version)

^a^Mixed model for adjustment of auto-correlation

^b^The values are given as the mean and the range in parenthesis

All kappa values >0.8 confirmed substantial intra- and interobserver reliabilities of the FCRa measurements.

## Discussion

We showed that there are differences in the accuracy of FCR in TKA based on the technique used in this study. Gap technique with tensor device of 20 lbf showed the most accurate and precise results. The measured resection technique with 3° external rotation and gap technique with spacer blocks showed a tendency of internal rotation in FCR. Postoperative 2 year clinical results were not significantly different among groups with different techniques for FCR.

Gap technique with the 89 N tensor device was the most accurate and precise method in obtaining the adequate FCR, but the ideal amount of tension has still not been determined. Asano et al. reported that the mean soft tissue tension was 126.8 N and 120.7 N in extension and flexion, respectively, in 77 knees. They concluded that 80–160 N was the appropriate tension, and tension in that range did not affect postoperative range of motion [14]. In their other report, the mean soft tissue tension in extension was 91.7 N in 64 knees, and the range of distribution was also very wide (approximately 55–175 N, not exactly revealed in the article, only revealed in the graph) [18]. Yoshino et al. reported that 86.1 N and 97.1 N in patellar eversion and reset, respectively, were the appropriate tensions in 25 PS-TKAs [15]. Lee et al. chose loads of 35 N for distraction force in their study [6] and Hanada et al. chose loads of 50 N for their study [7]. We used the 89 N tensor device in our study, and this value is similar to that documented in the above-mentioned reports.

In the measured resection group, 3° external rotation off the PCA was set based on previous reports [8, 9]. However, the FCRa was inconsistent and the percentage of outlier cases was high because of the variations in the extent of the posterior condylar erosion and position of the bony landmarks of each patient. Fehring et al. also reported higher rotational errors of at least 3° when FCR was determined by bony landmarks compared with the tension gap technique [16].

Several technical issues have been identified with the block placement method, which may be the cause of unsatisfactory implant placements. First, gap block could easily be rotated internally during the insertion or inevitably causes the femur to rotate externally because of the medial tightness in the varus knee, which applies to most of the participants in this study. This might have led to the internal rotation of femoral components in the block group. Second, the true tension of each participant might be underestimated. For example, if the true gap width of a patient is between 10 and 12.5 mm, it is evaluated uniformly as 10 mm because a thicker gap block cannot be inserted. Therefore, the block technique has a greater chance of inaccuracy. Some reports using gap block also introduced similar results although the causes were not explained clearly, that is, internal rotation of the femoral component [19].

The best reference for the FCR is still in debate. Several bony landmarks of the distal femur were proposed as the reference, including the PCA, cTEA, surgical transepicondylar axis (sTEA) and antero-posterior axis (Whiteside’s line). Fehring and Laskin found that the result was inferior to that of tensioned gap technique when the PCA was used as reference [16, 20]. Griffin also reported that the posterior condyles were potentially unreliable references [21]. Arima stated that Whiteside’s line was a reliable landmark in a valgus knee, and Whiteside reported that a better clinical outcome was observed in the group using Whiteside’s line as a reference compared with that using the PCA as a reference in a valgus knee [22, 23]. On the other hand, Nagamine’s study revealed that the PCA was more reliable than Whiteside’s line in knees with medial tibiofemoral arthritis [24]. Victor also reported that Whiteside’s line was least consistent on his CT-based kinematic study using cadavers [25]. Several studies concluded that the transepicondylar axis was more reliable than other landmarks in a typical varus knee with medial tibiofemoral arthritis. However, there are two transepicondylar axes (cTEA and sTEA), and several authors did not clearly state which of the two used in their studies [26–29]. Which of the two references is more appropriate and reproducible is unclear and there are several conflicting studies on this [28, 30, 31]. Based on our experience, we chose cTEA as a reference in this study.

This study has several limitations. First, the prosthesis and the instrument sets used for each group were different from one another. Although we think the type of prosthesis and instrument would not affect the FCRa significantly, different instruments could lead to different accuracy and precision. Second, because we chose the best cut image in axial CT with 1 mm thickness for measurement of FCRa rather subjectively, it could have not been the exact image to evaluate the FCRa of the case. However, we made it clear that axial CT cut was the best available image for FCRa measurement for the case. Third, the concept of FCR alignment in this study was based on mechanical alignment after TKA; meanwhile, there are several reports that patient-specific kinematic alignment is more important and more related to clinical outcome after TKA [32]. However, the focus of this study was to find the best method that can align the femoral component to the cTEA which the authors chose as the FCR reference based on literature reviews and personal experience. Fourth, surgeon errors could possibly occur with these three different techniques, due to each one having their own technical difficulties. Fifth, because this study was a single surgeon series, it is possible that the implementation of these three techniques could be different with different surgeons and the results could change if different surgeons were included in the study. However, this could also be an asset to this study that surgeon bias could be eliminated from the evaluation. And lastly, postoperative clinical evaluations were performed with 2-year postoperative data with less patients. Since the FCR could be a major factor to the long-term survival of the implant, longer follow-up clinical evaluations should be performed.

## Conclusions

Although there are both good and bad points in techniques for determining FCRs, the gap technique with the 20-lbf tensor device was the most accurate and precise method in obtaining adequate FCR. Measured resection with 3° external rotation and gap technique with blocks could lead to internal rotation of the femoral component. Postoperative 2 year clinical results were not significantly different among groups with different techniques for FCR.

## Abbreviations

CT: Computed tomography
cTEA: Clinical transepicondylar axis
FCR: Femoral component rotation
FCRa: Femoral component rotation angle
PCA: Posterior condylar axis
sTEA: Surgical transepicondylar axis
TKA: Total knee arthroplasty
